# Pharmacogenomic-guided opioid prescribing in hospice: reducing toxicity while maximizing symptom relief

**DOI:** 10.3389/fmed.2026.1920877

**Published:** 2026-09-02

**Authors:** Ben Thompson, Carrie Hyde, Binoy Singh

**Affiliations:** Gentiva, Atlanta, GA, United States

**Keywords:** end of life, hospice care, pain management, palliative care, pharmacogenomics, precision medicine, symptom management

## Abstract

Hospice care centers on interdisciplinary-driven, patient-focused, comprehensive and individualized care at the end of life. Effective pain management has been a cornerstone of hospice care, with up to 80% of patients experiencing moderate to severe pain at the end of life. Although central to hospice care, opioid prescribing faces major challenges: wide variability in response, frequent adverse effects (sedation, constipation, respiratory depression, cognitive impairment), and high rates of uncontrolled pain (20%–40% of patients inadequately relieved despite optimization). Traditional trial-and-error approaches are challenging in hospice due to short prognoses, complex comorbidities, and prevalent polypharmacy. Supportive evidence for the inclusion of Pharmacogenomics (PGx) testing into opioid prescribing is growing. Incorporating PGx testing into opioid prescribing practices at the end of life will face several hurdles including shifting protocols, cost of testing, and ethical concerns. With policy reforms, prescriber training, and rigorous research, PGx may become a useful tool in delivering truly personalized end-of-life care.

## Introduction

Hospice care embodies the essence of patient-centered medicine, prioritizing comfort, dignity, and quality of life for individuals facing terminal illnesses. As part of the Medicare Conditions of Participation for the hospice benefit, patients have the right to “[r]eceive effective pain management and symptom control from the hospice for conditions related to the terminal illness” (1). While effective pain control addresses total pain from an interdisciplinary perspective, including psychosocial and spiritual distress at the end of life, opioids are used as the primary therapeutic agents for alleviating moderate to severe physical pain, a symptom that affects up to 80% of hospice patients. Historically, opioids have been integral to palliative practices, evolving from morphine’s use in the 19th century to a diverse array of agents tailored to varying pain intensities (2).

The landscape of opioid prescribing in hospice in recent years is complex in the context of the opioid crisis, which has led to increased stigma around the use of opioid medications, increased regulatory scrutiny, and barriers to access. Opioids are associated with adverse effects and variability in analgesic response between patients as well.

Uncontrolled pain is prevalent among patients at the end of life. About 40% of patients dying in the hospital experience moderate to severe pain in the last 3 days of life (3). One study reported that 25.2% of caregivers reported inadequate pain management in 2015, increased from 15.5% in 2000 (4). Traditional opioid prescribing relies on empirical trial-and-error, which can prolong suffering while an appropriate opioid regimen is determined. In the population of frail, older adults, side effects such as sedation, constipation, respiratory depression, and cognitive impairment can become even more burdensome. The prevalence of uncontrolled pain in hospice underscores the urgency for innovation.

With over 500,000 deaths attributed to the opioid epidemic since 1999, policies aimed at curbing misuse have inadvertently restricted access for patients with pain at the end of life. A review of almost 300,000 Medicare fee-for-service decedents showed a decline in opioid prescribing at the end of life (38% MME decrease per decedent between 2007 and 2017) and increase in pain-related emergency department visits (5). One potential modality for personalizing opioid regimens is precision medicine via pharmacogenomics (PGx), a field that examines how genetic variations modulate drug responses. PGx tailors medication selection and dosing to individual genetic profiles, potentially mitigating risks of adverse events while enhancing efficacy in hospice settings where patients may have multiple comorbidities, polypharmacy (averaging 11–12 medications), and short life expectancies (6).

In hospice, opioids constitute 40%–80% of analgesic regimens, making PGx a promising strategy to reduce polypharmacy and deprescribe high-risk medications. Pilot studies incorporating pharmacogenetic data demonstrate that more than half of cases can suggest medication changes to improve efficacy and reduce the likelihood of adverse events. Key barriers to implementation include limited clinician familiarity with PGx and persistent equity gaps in access to testing. Potential solutions include interdisciplinary implementation as well as telehealth-enabled support for rural and underserved communities.

Key genes like CYP2D6, OPRM1, and COMT are responsible for opioid pharmacokinetics (absorption, distribution, metabolism, excretion) and pharmacodynamics (receptor binding and signaling). Despite PGx advancements in fields like oncology, where targeted therapies have revolutionized treatment, its adoption in palliative and hospice care remains nascent, hampered by limited evidence, implementation barriers, and ethical implementation. The end-of-life space is ripe for PGx adoption as rapid clinical decision making is crucial in this population. For instance, poor metabolizers of CYP2D6 may derive no benefit from codeine, while ultrarapid metabolizers risk life-threatening toxicity.

This paper synthesizes emerging evidence on PGx-guided opioid prescribing, delineates a hospice-tailored framework, delves into bioethical and psychosocial implications, and proposes policy and educational reforms. By promoting interdisciplinary coordination between clinicians, geneticists, ethicists, and policymakers, we provide a lens through which precision medicine can help address certain clinical challenges and align with the philosophy of hospice, providing whole-person care at the end of life.

### Pharmacogenomic mechanisms in opioid response

Understanding the pharmacology of opioids’ mechanism of action is crucial for appreciating PGx’s potential in hospice. The cytochrome P450 2D6 (CYP2D6) enzyme, encoded by the CYP2D6 gene on chromosome 22, is responsible for O-demethylation of prodrugs like codeine, tramadol, hydrocodone, and oxycodone into their active forms (morphine, O-desmethyltramadol, hydromorphone, and oxymorphone, respectively). CYP2D6 exhibits extensive polymorphism, with over 100 alleles classified into phenotypes: ultrarapid (UM), extensive (EM), intermediate (IM), and poor metabolizers (PM). Prevalence varies globally: PMs comprise 5%–10% of Caucasians, 1%–2% of Asians, and up to 20% in certain Ethiopian populations (7, 8). Approximately 15% of individuals have genetic variation in IM or PM status, and 3% are UMs (9).

For codeine, a prodrug, metabolism is critical for production of the active metabolite, morphine. PMs produce virtually no morphine, providing insignificant analgesia, while UMs generate supratherapeutic levels, increasing the risks of adverse effects like sedation and respiratory depression. Tramadol is metabolized by CYP2D6 to O-desmethyltramadol (M1) and has a dual mechanism of action (mu-opioid agonist plus serotonin and norepinephrine reuptake inhibitor). The FDA confirmed that M1 serum concentrations are 40% lower in PMs, intimating reduced analgesia.

Oxycodone presents a nuanced pharmacogenomic case because, unlike codeine or tramadol, the parent compound itself retains significant activity at the mu-opioid receptor. The primary metabolic pathway is CYP3A4-mediated N-demethylation to noroxycodone, while the CYP2D6-mediated demethylation to oxymorphone is the minor pathway. A phenomenon known as “physiologic shunting” occurs when a strong CYP3A4 inhibitor pushes the metabolic pathway towards the CYP2D6 enzyme. One study found that administration of ketoconazole, a strong CYP3A4 inhibitor, tripled the oxymorphone AUC while reducing noroxycodone AUC by 80% (10). A strong CYP3A4 inhibitor can significantly impact the metabolism and efficacy of oxycodone.

Methadone has one of the most pharmacogenetically complex profiles among opioids, with distinct genetic loci influencing metabolism, transport, and cardiac toxicity. Methadone has two enantiomers, (R)-methadone is responsible for methadone’s analgesic activity, while (S)-methadone contributes to cardiotoxicity. While historically CYP3A4 was thought to be the principal enzyme responsible for N-demethylation to its active metabolite, more recent evidence suggests that CYP2B6 is the primary determinant of methadone elimination (11). A 2015 study suggest that CYP2B6 polymorphism impacts (R)-methadone and (S)-methadone clearance (12).

Beyond metabolism, pharmacodynamic genes modulate receptor sensitivity. The Catechol-O-methyltransferase (COMT) enzyme is responsible for dopamine catabolism, affecting pain perception. A common COMT polymorphism, Val158Met has been demonstrated to impact pain response. Val/Val homozygotes exhibit lower pain thresholds, requiring increased opioid doses for effective analgesia (13).

There is a growing role of both pharmacokinetic and pharmacodynamic genes in opioid response variability. Identified enzymes encoded by these genes include CYP3A4 and CYP3A5 which are responsible for metabolism of fentanyl to norfentanyl and UGT2B7, which metabolizes morphine to morphine-3-glucuronide, its inactive metabolite (14).

The OPRM1 gene (located on chromosome 6q25.2) encodes the *μ*-opioid receptor. The most studied variant, A118G (rs1799971), substitutes aspartic acid for asparagine on the receptor, removing a glycosylation site and altering mu-opioid receptor binding affinity, with G-allele carriers requiring higher doses for equivalent analgesia. The carrier frequencies for the 118G allele are ~60% of Asian individuals, ~30% of white individuals, and 7% of African Americans. A 2019 meta-analysis revealed that carriers of the 118G allele required higher opioid doses for pain management and less nausea, especially in the postoperative setting (15). While there is robust and statistically significant evidence of increased analgesic needs of patients with the 118G allele, the size of the clinical effect is small. In a study of 1,000 patients undergoing breast cancer surgeries, AA homozygotes required 0.12 mg/kg of oxycodone in the postoperative period compared to 0.16 mg/kg of oxycodone for the GG homozygotes (16). The clinical relevance of OPRM1 testing remains limited, and no CPIC implementation guidelines exist for OPRM1-guided dosing (14).

Concerns for CYP2D6 phenotypic variation compound in patients at the end of life, where polypharmacy is common. Drug-gene (e.g., CYP2D6-SSRI inhibition) and drug–drug-gene conflicts amplify toxicity. A study of 100 hospice patients who underwent PGx testing found that only four patients had no drug-gene interactions and 16 had no drug–drug interactions (17). Commonly prescribed CYP2D6 inhibitors (bupropion, fluoxetine, paroxetine, duloxetine) can cause “phenoconversion,” functionally converting EMs to PMs (9). PGx panels assessing multiple pharmacogenes can identify actional drug-gene interactions in 30%–55% of patients at the end of life, with that number increasing when considering phenoconversion from polypharmacy.

### Evidence from literature

The foundation of evidence for PGx-guided prescribing in opioid therapy is expanding, especially in palliative contexts, though studies dedicated to the hospice population are limited. CYP2D6 variants’ role in metabolizing codeine and tramadol have been well described (18, 19). Poor metabolizers (PMs) show diminished conversion to active metabolites, resulting in decreased analgesic response, whereas ultrarapid metabolizers (UMs) experience amplified toxicity from excessive metabolite accumulation. CPY2D6 PMs and intermediate metabolizers (IMs) have more frequent ED visits for unmanaged pain than UMs or normal metabolizers (20). This finding suggests that incorporating data on *CYP2D6* genotype and accounting for drug interactions in opioid prescribing may improve pain management and reduce ED visits, which is important in individuals electing comfort only approaches at the end of life.

In hospice and palliative care, there is a high prevalence of medications that interact with PGx-implicated enzymes. A 2025 systematic review in BMJ Supportive and Palliative Care (11 studies, total *n* = 550) identified actionable PGx in up to 50% of cases, with an average of 4.6 drug-gene interactions per patient (21). Among 2,760 patients with cancer receiving medications for symptom management, 86% received a medication with PGx-indicated enzymes, and 84% of those cases involved CYP2D6 (22).

Recently the toll-like receptor (TLR4) pathway, a neuroimmune pathway, has emerged as a mediator of opioid-induced neuroinflammation, tolerance, and hyperalgesia. Opioids can non-stereoselectively activate TLr4 in the CNS, triggering a pathway leading to a neuroinflammatory response that can oppose opioid analgesia and contribute to the development of tolerance. In a rat model observing the ventrolateral periaqueductal gray locus, an area of the brain known to have morphine action, rats with inhibited TLR4 did not develop morphine tolerance (23). A multi-center Australian prospective cohort study of patients with advanced, incurable cancer (*n* = 54), eight statistically significant genetic associations, primarily in the TLR4 pathway, were determined between opioid dose, pain scores, and adverse opioid effects such as drowsiness and nausea. The implicated genetic associations included IL2, an inflammatory cytokine where variants had lower opioid doses and were eight times more likely to experience adverse effects due to opioids, although it was limited by a small sample size and insufficiently powered to analyze adverse effect and pain response by opioid type (24).

Pharmacist-centric analyses detail the wide variation of conversion of tramadol to morphine in various CYP2D6 phenotypes: a patient who is an IM given 80 mg of codeine has similar to an EM given 30 mg of codeine (25). The Clinical Pharmacogenetics Implementation Consortium (CPIC) guidelines recommend avoidance or alternatives for codeine and tramadol for PMs and UMs, while monitoring IMs closely (14).

The C-PAIN (Catalyzing Pharmacogenomic Analysis for Informing Pain Treatment) trial is a randomized prospective study examining the role of preemptive CYP2D6 genotyping in EHR-embedded decision support. Patients with metastatic solid tumors will undergo pharmacogenomic testing and then will be randomized to a PGx-guided arm or the control group. The primary outcome will be pain intensity at 45 days. Additionally, adverse effects, hospitalizations, morphine milliequivalents prescribed, and first opioid prescribed will be measured. The C-PAIN study is supported by a retrospective analysis of 61,572 patients with cancer, which linked IM/PM statuses to increased pain-related hospital encounters compared to patients with EM/UM status. They also found those with IM/PM status more frequently received opioid therapy with morphine and hydromorphone, which are not dependent on CYP2D6 (26).

Current testing for PGx-linked variants is mostly limited to blood draws and laboratory tests. A POC buccal swab testing CYP2C19 has been used to guide antiplatelet therapy recommendations with clopidogrel known to be less effective in patients with PM/IM status (9). This buccal swab has a median turnaround time of under 2 hours. CYP2D6 is substantially more complex given over 100 alleles have been identified, making miniaturization into a POC format more challenging. A 2026 study collected a subset of CYP2D6 and CYP2C19 variants to guide prescribing practices for patients with depression. This panel was sent to a laboratory with results typically reported within 7–10 days. Out of pocket single gene CYP2D6 tests can range in price from $200–$500, while PGx panels of multiple genes can cost upwards of $100. The average reimbursement rate for PGx claims from 2019 to 2021 was 46%, with panels reimbursed at a significantly higher rate (43% vs. 73%, *p* < 0.001) (27).

Identification of genes implicated in any aspect of opioid interaction, from the metabolic pathway to receptor signaling and modulation, highlights the potential of PGx to impact clinical decision making. Current literature supports the feasibility of PGx implementation and suggests potential benefits related to medication optimization and reduction in acute care visits. However, prospective hospice-specific trials remain essential to establish clinical efficacy, cost-effectiveness, and patient-centered outcomes.

### Hospice-specific framework

Integrating pharmacogenomics (PGx) into hospice requires a nuanced framework due to challenges of patients with short prognoses, family engagement with new interventions, and completing PGx panels in the home.

Economic models support the viability of PGx in hospice, projecting reductions in medication burden that may offset testing costs through fewer adverse drug events and unwanted hospitalizations. Sustainable expansion will likely require partnerships with laboratories to reduce assay costs.

Importantly, PGx aligns naturally with hospice’s interdisciplinary philosophy by personalizing symptom management while integrating into existing workflows, such as IDG meetings. Pharmacists can serve as PGx champions by interpreting results, educating clinicians, and supporting implementation. Tracking PGx-related outcomes and utilization metrics may demonstrate value to organizational leadership and support long-term program sustainability.

### Bioethical and psychosocial considerations

Integrating PGx into hospice opioid prescribing introduces complex ethical and psychosocial considerations rooted in principles of autonomy, beneficence, non-maleficence, and justice. These issues are especially pronounced in end-of-life care, where patients (and families) face declining decisional capacity, heightened vulnerability, and shift in focus from cure to comfort.

### Autonomy and informed consent

Respect for autonomy requires that patients, or when appropriate, their surrogates, make informed decisions regarding PGx testing. However, obtaining informed consent in hospice can be challenging due to delirium, emotional distress, and/or cognitive decline. Studies in palliative oncology suggest that PGx testing is highly acceptable when framed as a tool to improve symptom management, with reported acceptance rates exceeding 97% (28). Simplified, tiered consent processes that focus specifically on opioid management while offering optional research participation may help preserve autonomy without overwhelming patients or families. Advance care planning discussions and regular review can also support clearer alignment with patient goals and values.

### Beneficence and non-maleficence

The principle of beneficence supports PGx use when it improves analgesia and minimizes adverse effects like sedation, delirium, and overdose risk. Delirium alone affects an estimated 42%–88% of dying patients and can profoundly alter communication and meaningful family interaction (29). At the same time, non-maleficence requires careful attention to unintended harms, including delays in pain relief, anxiety related to genetic findings, or misinterpretation of results leading to undertreatment. Current recommendations emphasize pragmatic use of PGx only when results are likely to influence prescribing decisions. Broader concerns around privacy, discrimination, and informed consent, like in addiction medicine and pharmacogenetics literature, are equally relevant in hospice settings.

### Justice and equity

Equitable access to PGx testing remains a significant ethical concern. Hospice disparities already affect racial and ethnic minorities, rural populations, and economically disadvantaged patients (30). Because CYP2D6 metabolizer status varies significantly across populations, limited access to PGx testing could inadvertently widen existing disparities in pain management. Inclusive research participation, subsidized testing programs, and culturally sensitive counseling will be essential to ensure equitable implementation.

### Psychosocial and family considerations

For some patients, PGx-guided care may provide a sense of empowerment and personalization during an otherwise uncertain stage of life. However, genetic findings can also evoke guilt, anxiety, or family tension, particularly when hereditary implications arise. Interdisciplinary support involving psychologists, chaplains, and social workers may help address these concerns. Cultural and spiritual beliefs surrounding genetics may further influence perceptions, underscoring the importance of individualized communication. Registry data from palliative oncology suggest that many patients view PGx positively because it may “unlock” improved symptom relief. Experts recommend routine screening for emotional distress surrounding testing.

Family members, particularly adult children serving as surrogate decision makers, may struggle to balance comfort-focused goals against concerns about genetic implications. Support groups and spiritual care services may help normalize these conversations and address existential questions regarding suffering, inheritance, and meaning at the end of life. Institutional ethics committees may also play an important role in developing hospice-specific PGx protocols, including psychosocial assessment before and after treatment.

### Policy and educational considerations

Despite strong evidence supporting PGx guided opioid prescribing, adoption in hospice remains limited by reimbursement challenges, regulatory barriers, and clinician knowledge gaps. While CPIC guidelines provide actionable recommendations for opioids metabolized through CYP2D6 and related pathways, broader implementation will require coordinated policy and educational reform.

### Reimbursement reform

Medicare Hospice Benefit (Part A) coverage for PGx testing could improve access while potentially reducing downstream costs associated with adverse events and hospitalizations. Proposed hospice-specific PGx billing pathways could mirror reimbursement models already emerging in oncology, recognizing reductions in acute care utilization in hospice may offset panel costs.

### Regulatory modernization

Expanded FDA opioid labeling incorporating adult PGx warnings (e.g., codeine contraindication for PMs) and extending pediatric black-box alerts could improve prescribing safety. In parallel, integrating DEA-controlled substance monitoring processes may support more individualized prescribing while reducing scrutiny around opioid stewardship. HRSA – supported telehealth infrastructure and requirements for diverse representation in PGx guideline development could help address disparities and applicability.

### Educational initiatives

Educational initiatives are equally critical. Professional organizations such as the American Academy of Hospice and Palliative Medicine and National Alliance for Care at Home could develop targeted CME content addressing CPIC interpretation, ethical considerations, EHR integration, and practical prescribing workflows. Hospice certification renewal could eventually include foundational PGx competencies. Pharmacist-led “PGx rounds,” genetic counseling teleconsults, and the incorporation of PGx into palliative care fellowship curricula may further accelerate adoption. Patient-facing educational materials, including multilingual brochures and videos explaining “genetic pain codes,” could improve understanding and engagement.

Additional investment in research is also needed. Funding through organizations such as the National Institutes of Health and Patient-Centered Outcomes Research Institute could support randomized controlled trials evaluating PGx-guided care in hospice populations.

### Implementation challenges and solutions

One of the greatest barriers to integrating pharmacogenomics into hospice care is the lack of hospice-specific evidence. Most current data are extrapolated from oncology or chronic pain populations, with few randomized controlled trials conducted in hospice and palliative care settings. Future research should focus on pragmatic, cluster-randomized studies across multiple hospice organizations, measuring outcomes such as symptom burden, quality-adjusted life days, caregiver experience, and healthcare utilization.

Operational challenges also remain significant. Delays in home-based specimen collection, inconsistent turnaround times, and fragmented electronic health record systems can limit timely implementation. Emerging technologies such as point-of-care CYP2D6 testing platforms and EHR integration tools may help streamline workflows and improve clinical usability.

Clinician resistance presents another important obstacle. Many providers report limited familiarity with PGx interpretation and uncertainty about applying results in time-sensitive palliative care settings. Educational strategies such as pharmacist-led “train-the-trainer” programs, case-based learning, and audit-feedback models demonstrating successful PGx-guided outcomes may improve clinician confidence and adoption.

Financial concerns also influence implementation. While upfront testing costs may create hesitation, value-based reimbursement models tied to reduced adverse drug events, medication optimization, and healthcare utilization could improve sustainability and payer support over time.

Equity disparities must also be addressed thoughtfully. Minority populations remain underrepresented in many pharmacogenomic databases, and rural communities often face limited access to testing infrastructure. Community health worker-led outreach initiatives, telehealth-enabled counseling, and ancestry-informed PGx algorithms may help reduce bias and improve equitable access to precision medicine approaches.

Finally, data privacy and genetic information security remain critical considerations. HIPAA-compliant cloud platforms, patient-controlled access to genomic information, and de-identified research repositories will be essential to maintaining trust and protecting sensitive data.

### Future directions and research agenda

The future of hospice and palliative care PGx will likely be shaped by advances in technology, predictive analytics, and personalized medicine. Artificial intelligence-driven clinical prediction models may eventually combine PGx data with clinical variables to guide opioid selection and dosing in real time. Wearable biosensors capable of monitoring physiologic opioid response and symptom burden could further enhance individualized care.

Expanding PGx testing panels beyond common CYP variants may also improve precision. Future research will likely incorporate rare genetic variants, pharmacodynamic markers, pseudoconversion in the face of polypharmacy, microbiome influences on drug metabolism, and epigenetic factors associated with aging and serious illness.

Additional work is needed in historically understudied populations, including Hispanic, Indigenous, and geriatric patients, to ensure PGx recommendations are both accurate and equitable across diverse groups.

Emerging digital therapeutics may further complement PGx-guided care. Future integration may include personalized virtual reality pain management programs, chatbot-supported symptom reporting, and AI-assisted clinical decision support tools.

Collectively, these initiatives position hospice and palliative care PGx at the forefront of precision palliative medicine. Continued investment in research, infrastructure, and implementation science will be essential to realizing its full potential in improving comfort, safety, and quality of life for patients with serious illness.

### Limitations of pharmacogenomics at end of life

While this paper has discussed the significance of pharmacogenomics and the potential implications of precision medicine at the end of life, there are some limitations. Variants at the CYP2D6 enzyme have been most studied in opioid prescribing. Codeine and tramadol are most dependent on this enzyme and much less utility in analgesia in patients at the end of life than other opioids like morphine, fentanyl, and hydrocodone. Oxycodone is metabolized by CYP2D6, but this is a minor pathway with CYP3A4 implicated in the major pathway. Perhaps the neuroinflammatory TLR4 pathway or the genes encoding the mu-opioid receptor will emerge as more clinically significant over time.

## Conclusion

Pharmacogenomic-guided opioid prescribing could impact the landscape of opioid stewardship in hospice and palliative medicine, using precision to navigate the intricacies and personalize pain management. Genetic-informed prescribing can reduce toxicity while reducing the time to the appropriate medication regiment, with actionable alleles in 50% of the population. With the development of rapid assays and EHR alerts and consideration of bioethical principles, pharmacogenomic-based prescribing can improve the care of patients and families at the end of life.

In the future, widespread uptake of pharmacogenomics in healthy patients may further decrease the financial hurdles of implementation of genetic sequencing of patients at the end of life.

Changes to education and policy, including reimbursement, regulatory augmentations, and clinical competencies will prepare the field for the inevitable technological leaps that will promote equity in the field. Integrating PGx into hospice and palliative care is the embodiment of patient-centered care, with personalized opioid prescribing based on the genetic material that is unique to each patient.

## References

[1] eCFR (2026) Code of Federal Regulations. Hospice Conditions of Participation. Available online at: https://www.ecfr.gov/current/title-42/chapter-IV/subchapter-B/part-418/subpart-C/section-418.52 (Accessed June 26, 2026)

[2] Ballantyne JC Mao J. Opioid therapy for chronic pain. N Engl J Med. (2003) 349:1943–53. doi: 10.1056/NEJMra025411, 14614170

[3] Blinderman CD Billings JA. Comfort care for patients dying in the hospital. N Engl J Med. (2015) 373:2549–61. doi: 10.1056/NEJMra1411746, 26699170

[4] Teno JM Freedman VA Kasper JD Gozalo P Mor V. Is care for the dying improving in the United States? J Palliat Med. (2015) 18:662–6. doi: 10.1089/jpm.2015.0039, 25922970 PMC4532900

[5] Enzinger AC Ghosh K Keating NL Cutler DM Landrum MB Wright AA. US trends in opioid access among patients with poor prognosis cancer near the end-of-life. J Clin Oncol. (2021) 39:2948–58. doi: 10.1200/JCO.21.00476, 34292766 PMC8425843

[6] Tjia J Alcusky M Rumbut J Furuno JP. Medications reported to the medicare hospice program after medicare drug policy changes, 2014–2018. J Pain Symptom Manag. (2025) 70:e55–64. doi: 10.1016/j.jpainsymman.2025.03.031, 40187379 PMC12150345

[7] Wilkinson GR. Drug metabolism and variability among patients in drug response. N Engl J Med. (2005) 352:2211–21. doi: 10.1056/NEJMra032424, 15917386

[8] Pratt VM Cavallari LH Del Tredici AL Gaedigk A Hachad H Ji Y . Recommendations for clinical CYP2D6 genotyping allele selection. J Mol Diagn. (2021) 23:1047–64. doi: 10.1016/j.jmoldx.2021.05.013, 34118403 PMC8579245

[9] Cavallari LH Myers RA Chakraborty H Skaar TC Gray CF Baye JF . CYP2D6-guided opioid management and postoperative pain control: a randomized clinical trial. JAMA Netw Open. (2026) 9:e2558299. doi: 10.1001/jamanetworkopen.2025.58299 41719044 PMC12924106

[10] Samer C Daali Y Wagner M Hopfgartner G Eap C Rebsamen M . The effects of CYP2D6 and CYP3A activities on the pharmacokinetics of immediate release oxycodone. Br J Pharmacol. (2010) 160:907–18. doi: 10.1111/j.1476-5381.2010.00673.x, 20590587 PMC2935997

[11] Ahmad T Valentovic MA Rankin GO. Effects of cytochrome P450 single nucleotide polymorphisms on methadone metabolism and pharmacodynamics. Biochem Pharmacol. (2018) 153:196–204. doi: 10.1016/j.bcp.2018.02.020, 29458047 PMC6148365

[12] Kharasch ED Regina KJ Blood J Friedel C. Methadone pharmacogenetics. Anesthesiology. (2015) 123:1142–53. doi: 10.1097/ALN.0000000000000867, 26389554 PMC4667947

[13] Rakvåg TT Klepstad P Baar C Kvam T-M Dale O Kaasa S . The VAL158MET polymorphism of the human catechol-O-methyltransferase (COMT) gene may influence morphine requirements in cancer pain patients. Pain. (2005) 116:73–8. doi: 10.1016/j.pain.2005.03.032, 15927391

[14] Magarbeh L Gorbovskaya I Le Foll B Jhirad R Müller DJ. Reviewing pharmacogenetics to advance precision medicine for opioids. Biomed Pharmacother. (2021) 142:112060. doi: 10.1016/j.biopha.2021.112060, 34523422

[15] Chu H. The relevance of the oprm1 118A>g genetic variant for opioid requirement in pain treatment: a meta-analysis. Pain Physician. (2019) 4:331–40. doi: 10.36076/ppj/2019.22.331 31337162

[16] Cajanus K Kaunisto MA Tallgren M Jokela R Kalso E. How much oxycodone is needed for adequate analgesia after breast cancer surgery: effect of the oprm1 118A>G polymorphism. J Pain. (2014) 15:1248–56. doi: 10.1016/j.jpain.2014.09.002, 25239082

[17] Bull JH Bice T Satterwhite WJ Massie L Burpee E Knotkova H . Feasibility and acceptability of a pharmacogenomic decision support system in palliative care. J Palliat Med. (2022) 25:219–26. doi: 10.1089/jpm.2021.0270, 34714127

[18] Stamer UM Musshoff F Kobilay M Madea B Hoeft A Stuber F. Concentrations of tramadol and O-desmethyltramadol enantiomers in different CYP2D6 genotypes. Clin Pharmacol Ther. (2007) 82:41–7. doi: 10.1038/sj.clpt.6100152, 17361124

[19] Smith HS. Opioid metabolism. Mayo Clin Proc. (2009) 84:613–24. doi: 10.1016/S0025-6196(11)60750-7, 19567715 PMC2704133

[20] Nahid NA McDonough CW Wei Y-JJ Gong Y Empey PE Haddad A . CYP2D6 phenotypes and emergency department visits among patients receiving opioid treatment. JAMA Netw Open. (2025) 8:e2523543. doi: 10.1001/jamanetworkopen.2025.23543, 40720122 PMC12305384

[21] Barry C Patel M. Pharmacogenomics and symptom management in palliative and supportive care: a scoping review. BMJ Support Palliat Care. (2025) 15:158–67. doi: 10.1136/spcare-2024-005205 39805678

[22] Patel JN Boselli D Jandrisevits EJ Hamadeh IS Salem A Meadors P . Potentially actionable pharmacogenetic variants and symptom control medications in oncology. Support Care Cancer. (2021) 29:5927–34. doi: 10.1007/s00520-021-06170-4, 33758969

[23] Eidson LN Murphy AZ. Blockade of toll-like receptor 4 attenuates morphine tolerance and facilitates the pain relieving properties of morphine. J Neurosci. (2013) 33:15952–63. doi: 10.1523/JNEUROSCI.1609-13.2013, 24089500 PMC3787504

[24] Wong AK Klepstad P Somogyi AA Vogrin S Le B Philip J . Effect of gene variants on opioid dose, pain and adverse effect outcomes in advanced cancer: an explorative study. Pharmacogenomics. (2009) 24:901–13. doi: 10.2217/pgs-2023-0207 38126330

[25] Yang Y Zhang X Wang Y Xi H Xu M Zheng L. Physiologically based pharmacokinetic modeling to predict the pharmacokinetics of codeine in different CYP2D6 phenotypes. Front Pharmacol. (2024) 15:1342515. doi: 10.3389/fphar.2024.1342515, 38756374 PMC11096448

[26] Reizine N Danahey K Schierer E Liu P Middlestadt M Ludwig J . Impact of cyp2d6 pharmacogenomic status on pain control among opioid-treated oncology patients. Oncologist. (2021) 26:e2042–52. doi: 10.1002/onco.13953, 34423496 PMC8571740

[27] Lemke LK Alam B Williams R Starostik P Cavallari LH Cicali EJ . Reimbursement of pharmacogenetic tests at a tertiary Academic Medical Center in the United States. Front Pharmacol. (2023) 14:1179364. doi: 10.3389/fphar.2023.1179364, 37645439 PMC10461057

[28] Cuffe S Hon H Qiu X Tobros K Wong C-KA De Souza B . Cancer patients’ acceptance, understanding, and willingness-to-pay for pharmacogenomic testing. Pharmacogenet Genomics. (2014) 24:348–55. doi: 10.1097/FPC.0000000000000061, 24911662

[29] Watt CL Momoli F Ansari MT Sikora L Bush SH Hosie A . The incidence and prevalence of delirium across palliative care settings: a systematic review. Palliat Med. (2019) 33:865–77. doi: 10.1177/0269216319854944, 31184538 PMC6691600

[30] Ornstein KA Roth DL Huang J Levitan EB Rhodes JD Fabius CD . Evaluation of racial disparities in hospice use and end-of-life treatment intensity in the regards cohort. JAMA Netw Open. (2020) 3:e2014639. doi: 10.1001/jamanetworkopen.2020.14639, 32833020 PMC7445597

